# Lymphocytes of Patients with Alzheimer’s Disease Display Different DNA Damage Repair Kinetics and Expression Profiles of DNA Repair and Stress Response Genes

**DOI:** 10.3390/ijms140612380

**Published:** 2013-06-10

**Authors:** Giovana S. Leandro, Romulo R. Lobo, Douglas V. N. P. Oliveira, Julio C. Moriguti, Elza T. Sakamoto-Hojo

**Affiliations:** 1 Department of Genetics, Faculty of Medicine of Ribeirão Preto, University of São Paulo, Ribeirão Preto, S.P. 14049-900, Brazil; E-Mails: giovanasl@usp.br (G.S.L.); dodausp@gmail.com (D.V.N.P.O.); 2 Department of Medical Clinic, Faculty of Medicine of Ribeirão Preto, University of São Paulo, Ribeirão Preto, S.P. 14049-900, Brazil; E-Mails: rrlobo22@yahoo.com.br (R.R.L.); moriguti@fmrp.usp.br (J.C.M.); 3 Department of Biology, Faculty of Philosophy, Sciences and Letters of Ribeirão Preto, University of São Paulo, Ribeirão Preto, S.P. 14040-901, Brazil

**Keywords:** Alzheimer’s disease, DNA repair, oxidative stress

## Abstract

Alzheimer’s disease (AD) is a progressive neurodegenerative disorder, characterized by loss of memory and cognitive capacity. Given the limitations to analyze brain cells, it is important to study whether peripheral lymphocytes can provide biological markers for AD, an interesting approach, once they represent the overall condition of the organism. To that extent, we sought to find whether lymphocytes of AD patients present DNA damage and repair kinetics different from those found in elderly matched controls (EC group) under *in vitro* treatment with hydrogen peroxide. We found that AD patient cells indeed showed an altered DNA repair kinetics (comet assay). Real-time quantitative analysis of genes associated with DNA stress response also showed that *FANCG* and *CDKN1A* are upregulated in AD, while *MTH1* is downregulated, compared with the control group. In contrast, the expression of *ATM*, *ATR* and *FEN1* genes does not seem to differ between these groups. Interestingly, TP53 protein expression was increased in AD patients. Therefore, we found that kinetics of the stress response in the DNA were significantly different in AD patients, supporting the hypothesis that repair pathways may be compromised in AD and that peripheral lymphocytes can reveal this condition.

## 1. Introduction

Alzheimer’s disease (AD) is a progressive and neurodegenerative brain disorder, characterized by the loss of memory and cognitive capacity, being severe enough to interfere with daily function and quality of life [1]. Alois Alzheimer first described the disease in 1907 [2]; although many studies have shown metabolic and oxidative abnormalities as early key factors involved in AD development, the molecular basis underlying its pathophysiology remains controversial [3,4]. Usually, there are two forms of AD: the early-onset familial AD (approximately 5% of the cases) and the late-onset or sporadic AD (95% of the cases) [2,5]. In the familial AD, the disease appears before the age of 60 years, and some studies suggested that it is connected to rare and highly penetrating mutations mainly in three genes, namely the amyloid precursor protein (APP), the presenilin 1 (PS1) and the presenilin 2 (PS2) genes [6–10]. The late onset, or sporadic AD, seems to have a more complex cause and less apparent familial aggregation [5]. Susceptibility to late-onset AD may be conferred by numerous genetic risk factors of relatively high frequency, but low penetrance and, therefore, small effect size, and it is important to emphasize that up to 60%–80% of sporadic AD should be genetically determined [11]. However, environmental and epigenetic factors may also be important for determining an individual risk, although the precise nature and mechanisms underlying these components remain largely elusive [5].

It has been shown that cells from individuals with AD present deficiencies in the repair of DNA lesions, suggesting the involvement of DNA repair processes in the pathogenesis of the disease [12–14]. Evidence for increased DNA damage in AD includes high levels of oxidative damage, observed in both nuclear and mitochondrial DNA isolated from brain regions of AD patients [15], increased markers of oxidative DNA damage in peripheral tissues [13,16] and enhanced levels of mtDNA deletions and point mutations [17]. Furthermore, oxidative stress can cause modifications in organic molecules, such as RNA, proteins and DNA that are thought to play a key role in the selective neuronal loss associated with aging and in the pathogenesis of neurodegenerative diseases, such as AD [18–20]. Despite many reports indicating the involvement of oxidative stress in AD, it is still unknown whether it is the cause or consequence of the disease.

Considering the difficulty to analyze brain cells, it has been suggested that peripheral lymphocytes can provide biological markers to study the pathophysiology of AD [21,22]. Although lymphocytes are highly specialized cells, they can represent the overall condition of the organism, since they circulate through the whole body and interact with several tissues [23]. For instance, assessment of oxidized bases in the DNA of lymphocytes has already been reported as the means to evaluate oxidative stress in the body [23]. Moreover, the regulation of proliferation and differentiation of lymphocytes involves signal transduction pathways, similar to those occurring in neurons and lymphocytes still expressing a variety of cell surface receptors comparable to neurons [24]. There are several disease-specific changes reported in the immune system of patients with AD [25], suggesting that AD is a systemic disease that affects many organs in the organism, although clinical effects occur primarily in the brain tissue [26]. Hence, it is important to investigate the efficiency of the DNA repair system in lymphocytes of patients with AD.

In the present work, we sought to evaluate the levels of DNA damage and repair kinetics in lymphocytes of AD and elderly control (EC) individuals, analyzed under the same experimental condition. To test the hypothesis that DNA repair capacity might be compromised in AD, lymphocytes were collected from both groups of individuals and then exposed to hydrogen peroxide (H_2_O_2_). Furthermore, we also assessed the expression levels of several genes associated with DNA repair and stress responses, such as *ATM*, *ATR*, *FANCG*, *FEN1*, *CDKN1A*, *MTH1*, *SOD1* and *TP53* genes, compared with age-matched control individuals. These alterations found in lymphocytes can be relevant and need to be further investigated to search for biomarkers that may characterize the disease.

## 2. Results and Discussion

**Figure 1:**
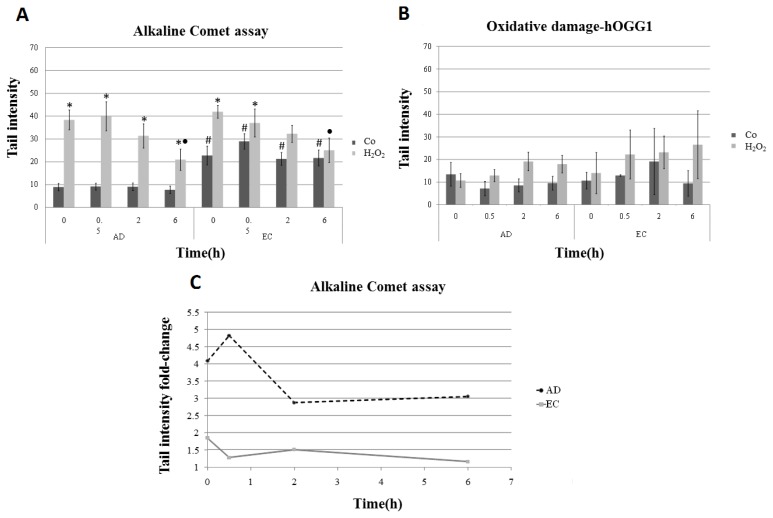
DNA damage measured as tail intensity (% of DNA in the tail) in the comet assay. Lymphocyte cultures from AD and EC groups of individuals were submitted to treatment with hydrogen peroxide (H_2_O_2_) for 1 h and harvested at different recovery times (0, 0.5, 2 and 6 h) after treatment. (**A**) DNA damage analyzed by alkaline comet assay; (**B**) estimated net amount of oxidative damage: each value of tail intensity obtained in the conventional comet assay (without hOGG1 enzyme) was subtracted from that observed in the assay performed with the addition of hOGG1; (**C**) scatterplot showing the extent of DNA damage presented by treated samples in relation to the control samples; fold-change was calculated using mean values of tail intensity. * Statistically significant difference between each treatment with H_2_O_2_, and its respective control (*p <* 0.05); ● Statistically significant difference between the mean values of tail intensity obtained at 0.5, 2 and 6 h of recovery relative to the corresponding initial time (0 h) (*p <* 0.05); # Statistically significant difference when the EC sample was compared with the corresponding AD sample (*p <* 0.05).

**Figure 2:**
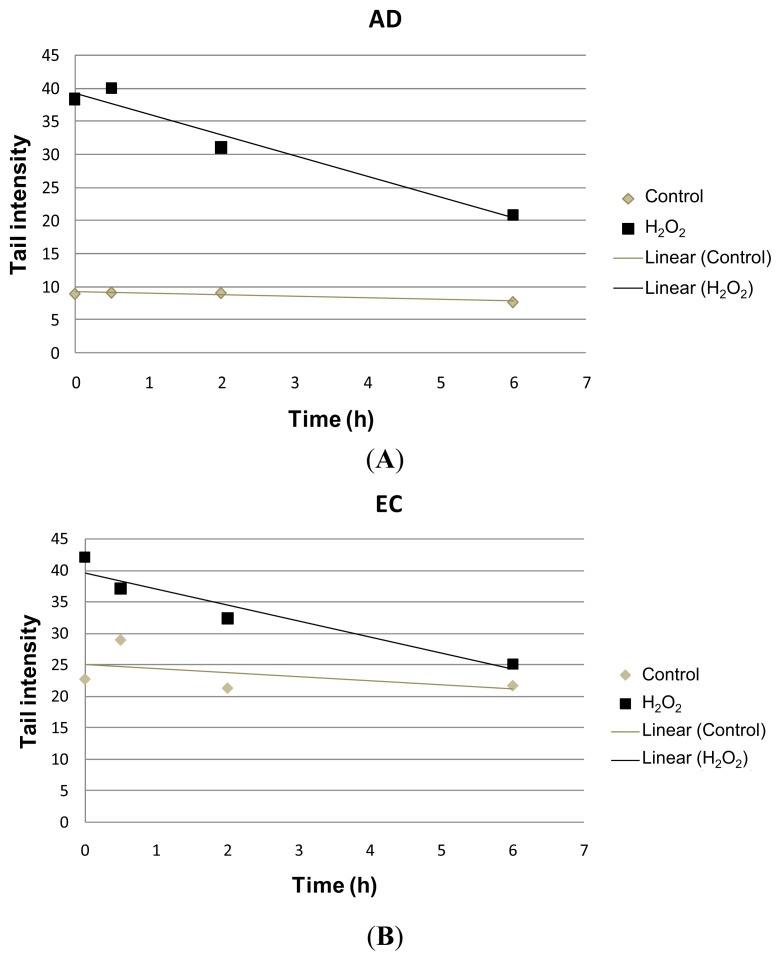
Scatter plots with mean values of tail intensities as a function of time obtained in lymphocyte cultures of AD patients (*R*^2^ = 0.9564) treated with H_2_O_2_ and the respective EC group (*R*^2^ = 0.9178), as analyzed by linear regression. Time-dependent repair kinetics (decrease in the values of tail intensities) displayed by lymphocytes of (**A**) AD patients and (**B**) EC individuals treated in culture with H_2_O_2_.

The present study assessed hydrogen peroxide-induced DNA damage, as well as the repair kinetics in lymphocytes of individuals affected by AD, compared with the EC group, by using the alkaline comet assay, both the conventional method and the modified version with the hOGG1 enzyme treatment. Lymphocytes from both groups of individuals were analyzed after 1 h exposure to H_2_O_2_, and samples were harvested at different recovery times: 0, 0.5, 2 and 6 h. Surprisingly, untreated cells showed lower levels of DNA damage, measured as tail intensity (TI), in the AD group (TI = 8.96), when compared with the respective control (TI = 22.73) (Table 1, Figure 1A). However, in terms of magnitude, the induction of DNA damage by H_2_O_2_ treatment was higher in the AD group, with a four-fold increase in tail intensity when compared to its mock-treatment counterpart, whereas in the EC group, a two-fold increase was observed (Figure 1C). Although the tail intensities (AD = 38.40 and EC = 41.99) did not significantly differ between groups, the ANOVA statistical test applied to the results showed significant differences (*p <* 0.05) in the repair kinetics between the two groups of AD and EC individuals (Figure 1A).We also observed a time-dependent decrease (*R*^2^ = 0.9564, *p =* 0.0221 and *R*^2^ = 0.9178, *p =* 0.0420, obtained for AD and EC groups, respectively) in the values of tail intensities in lymphocytes treated with H_2_O_2_ (Figure 2). Following recovery times, the tail intensities gradually decreased until 6 h, although AD patients did not completely recover, and damage levels at 6 h were approximately three-times higher in the AD compared to the EC group (Figure 1C). These results might indicate that repair capability seems to be reduced in AD patients.

Leutner *et al.* [27] reported that the basal levels of DNA damage observed in lymphocytes of AD patients were higher, compared with the control group, and this is in contrast to the results obtained in the present study, in which the amount of basal damage in elderly controls was greater than that observed in AD patients. There are few explanations for this observation. One possibility to explain this divergence is the different experimental condition, mainly regarding the time in which cells were evaluated in both studies. Leutner *et al.* [27] analyzed DNA damage immediately after blood collection, while in the present work, cells were cultured for 48 h. Furthermore, AD patients are chronically exposed to a high metabolically-derived background level of ROS generated in the organism by many sources, among which are mitochondrial, amyloid-β (Aβ) and advanced glycation end products—AGEs [28]. In our study, we need to consider the *in vitro* condition of our experiments with lymphocytes, conducted in the absence of *in vivo* stress conditions under which the organisms of AD patients have been exposed; cells from AD and EC individuals were cultured under the same conditions to check DNA damage and repair kinetics by the comet assay. Moreover, Collins [23] and Torbergsen *et al*. [29] observed that immediately after isolation, human lymphocytes suffer oxidative damage from sudden exposure to the high concentration of O_2_ in the atmosphere, and possibly, these aspects may provide at least some contribution for the above mentioned discrepancies between the results.

Another explanation refers to a compensatory mechanism in the organism of patients with AD, as suggested in the literature [30,31] and on the basis of several reports about the role of oxidative stress in neurodegeneration observed in AD patients [13,28,32–37]. The increase of oxidative damage in the DNA of AD patients might be due to a decrease in antioxidant defense mechanism [38], as well as to the weakening of a specific repair system towards the repair of oxidized guanine [39]. There is also evidence that the increase in 8-oxo2dG occurs much earlier than clinical symptoms [13,28]. However, according to Dezor *et al.* [30], 8-oxo2dG levels in peripheral blood lymphocytes of AD patients depend on the degree of dementia and suffer an increase as it progresses from mild to moderate, with a tendency to remain steady in severe depression, as evaluated by the Mini-Mental State Examination score. This phenomenon probably occurs as a consequence of a compensatory system, which can be activated in the organism of patients [30,31], somehow leading to the prevention of oxidative stress or reduction in metabolic activity, especially in advanced stages of the disease, as it might be the situation with patients in the present study; most of them were categorized as being in the moderate to advanced stage of the disease (Table 2); and probably, this could explain, at least in part, the results regarding the lower levels of DNA damage in AD relative to the EC group.

In AD patients, besides the hypothesis of a compensatory effect mentioned above, there is evidence that lymphocytes of patients cultured with PHA show altered cell cycle progression after 48 h of culture [22,40,41]. Thus, it can be speculated that repair sites might be reduced in consequence of altered cell cycle progression or deficiency in repair capacity. The standard alkaline comet assay performed in this study detects DNA strand breaks and alkali-labile sites [23]. These alkali-labile sites include apurinic and apyrimidinic sites, or AP sites, which arise from the loss of damaged bases, leaving a base-less sugar in the backbone. AP sites in DNA structure also occur as intermediates during base excision repair (BER) and may also arise spontaneously, owing to altered chemical stability, resulting from changes in bases or sugars [23,42]. Therefore, based on this information, it can be suggested that higher amounts of DNA damage found in untreated EC, compared with AD patients, correspond to intermediate sites of DNA repair; by assuming that cells from EC individuals may present a cell cycle progression and metabolic activity close to normal rates, which can generate oxidized bases in the DNA. In contrast, the opposite may occur in AD: low metabolic rates and deficiency in cell proliferation might reduce the amount of DNA damage in AD compared with EC, in addition to the lower formation of base excision repair sites.

Therefore, the information available in the literature supports the assumption that low levels of DNA damage observed in AD (untreated samples) might be a consequence of a compensatory system, especially in patients with advanced stages of the disease, and also to deficiencies in cellular processes, such as DNA repair, proliferation and metabolism.

Moreover, differences observed between AD and EC groups regarding the responses to the induction of DNA damage by H_2_O_2_ indicate that EC individuals present a more efficient mechanism by which cells are capable of minimizing the effects of H_2_O_2_. This protection has been associated with antioxidant mechanisms [43,44] or DNA repair capacity [45].

The results obtained in the comet assay with hOGG1 enzyme treatment showed increased values of tail intensities, even regarding the basal levels for AD (TI = 17.32) and EC (TI = 24.10) groups (Table 1), compared with the results obtained in the conventional alkaline comet assay. To estimate the amount of oxidative damage, each value of TI (obtained for each sample analyzed by the conventional comet assay, without hOGG1 enzyme) was subtracted from that observed in the assay with the addition of hOGG1. The values obtained corresponded to the net amount of oxidative damage caused by the H_2_O_2_, thus sorting out the amount of DNA breaks, which were detected by the conventional comet assay. This calculation showed a lack of significant difference between AD and EC groups regarding the hydrogen peroxide-induced oxidative DNA damage (Figure 1B). Curiously, the total amount of induced-DNA damage (DNA breaks and oxidative damage) obtained in the comet assay with hOGG1 was not significantly reduced in the EC group, even after 6 h of recovery, while the AD group showed a significant decrease (44.47 to 31.59) (*p <* 0.05), even though these values did not reach control levels (Table 1).

Kadioglu *et al.* [16] employed the comet assay with two restriction endonucleases (Endo III and Fpg) and observed increased oxidative DNA damage in lymphocytes of AD patients, suggesting the possibility of increased free radical damage associated with the disease. Similarly, Morocz *et al.* [37] found high concentrations of the oxidized base 8OHdG in lymphocytes of patients with AD compared with controls of similar age, under conditions of treatment with 150 μM H_2_O_2_. The authors analyzed oxidative damage immediately after lymphocyte collection. Possibly, the increased amount of basal levels of oxidized bases could be due to the presence of endogenous oxidative stress or the inability to maintain the redox balance in AD patients. Thus, endogenous oxidative stress could be a crucial factor in the observed differences between the results, although other factors, such as clonal selection of cultured lymphocytes, could also influence the results, but this assumption should be further investigated.

In this study, it is possible that low levels of the net amount of oxidative DNA damage (Figure 1B) can also be a consequence of the period of treatment with H_2_O_2_. Inside the cells, H_2_O_2_ is broken into high reactive molecules with short half-lives [46,47], which also cause DNA damage [48]. One-hour H_2_O_2_ treatment could cause, at the first moment, an induction of oxidative damage, followed by a short period of recovery/DNA repair, thus masking the real effect of the chemical.

DNA damage evaluated by the conventional alkaline comet assay also showed that AD patients, when compared with the EC group, present impairment in repair kinetics, since after 6 h of recovery, the AD group did not reach the basal levels of DNA damage, as observed in the EC group. However, in the comet assay with hOGG1, we did not observe a significant reduction in DNA damage levels for either group, even after 6 h of recovery. Thus, in spite of the limitations imposed by the number of individuals sampled in the present study, the comparison of DNA damage responses and repair kinetics displayed by AD patients indicated that repair capacity might be compromised in AD.

Considering that DNA repair kinetics showed differences between AD and EC groups, we studied expression profiles of several genes (*ATM*, *ATR*, *FANCG*, *FEN1*, *CDKN1A*, *MTH1*, *SOD1* and *TP53*), whose functions are related to DNA repair and responses to stress.

**Figure 3:**
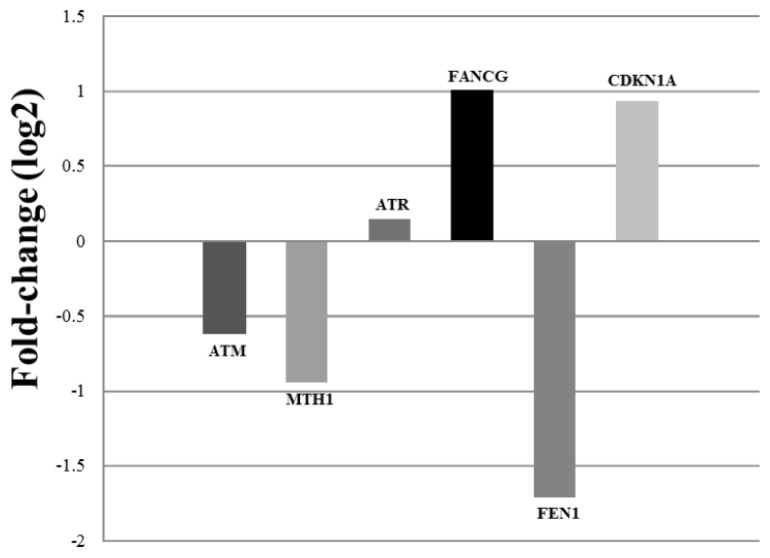
Relative gene expression (fold-change) for *ATM*, *MTH1*, *ATR*, *FANCG*, *FEN1* and *CDKN1A* genes in lymphocytes of AD patients compared with elderly matched control subjects analyzed by qRT-PCR. *HPRT1*, *GUSB* and *B2M* were used as endogenous controls.

Analysis of transcript expression by real-time qPCR showed an upregulation of *FANCG* and *CDKN1A* genes in lymphocytes from AD patients (Figure 3). *FANCG* belongs to the group of Fanconi Anemia (FA) genes. The FA proteins, FANC-A, B, C, E, F, G and L, assemble in a nuclear complex (FA complex) required for activation through monoubiquitination of FANCD2 and FANCI in response to DNA damage [49,50]. It was demonstrated that the FANCG protein could have an important function in repair and protection against oxidative damage [51–53]. After DNA damage induction, ATM [54] and ATR are required for FANCD2 phosphorylation, and ATR-dependent phosphorylation is essential for the correct monoubiquitination of FANCD2 on lysine 561 (K561) [55,56]. Probably, the upregulation of this gene in AD individuals may be associated with the chronic condition of oxidative stress to which lymphocytes have been submitted. Indeed, the absence of differences in the expression levels of ATM and ATR genes may suggest a blockage in the FA pathway that deserves to be further studied.

The *CDKN1A* gene encodes a protein member of cyclin-dependent kinases inhibitors (CDKN1A). This protein regulates the transition between the G1 and S-phase by inhibiting the activity of cyclin D-CDK4, cyclin E-CDK2 and cyclin A-CDK2 [57–60]. The transcriptional induction of this gene is regulated by TP53-dependent and non-dependent pathways [61]. Although the role of *CDKN1A* is well-known, the mechanisms underlying its regulation are still unclear. Thus, the induction of CDKN1A transcription in lymphocytes, as demonstrated in the present study, might be associated with high levels of stress in lymphocytes from AD patients, reinforcing the involvement of the TP53 pathway. This is compatible with the higher expression of TP53 in the AD group compared with EC individuals observed in the present study.

In contrast, we observed a downregulation of the *MTH1* gene in lymphocytes from AD patients (Figure 3). The MTH1 protein can play an important role in the maintenance of the fidelity of DNA replication and transcription, thus protecting the organism from cancer development and neuronal degeneration [62]. It has been established that 8-oxo-dGTP, an oxidized form of dGTP, represents the main endogenous source of spontaneous mutations [63–65], which can be eliminated by several repair pathways. The MTH1 protein, an ortholog of MutT in *Escherichia coli*, is important to hydrolyze 8-oxo-dGTP to its monophosphate form, thus eliminating altered substrates from the DNA precursor pool [65]. The MTH1 protein efficiently hydrolyzes 8-oxo-GTP, minimizing errors caused by misincorporation of oxidized guanine nucleotides into RNA [64,66,67]. The expression of the MTH1 gene in human tissues is upregulated during proliferative activation of cells or by oxidative stress [68]. Song *et al.* [69] showed increased amounts of 8-oxoguanine in the RNA and decreased expression of *MTH1* in the hippocampus of AD patients, suggesting that *MTH1* deficiency might be a causative factor for aging and age-related disorders. Therefore, the downregulation of this gene in AD patients might be associated with the repair deficiency in response to oxidative damage stimulus. This is an interesting aspect to be further investigated.

The *FEN1* gene did not show significant alteration in expression profiles displayed by AD patients (Figure 3), relative to controls. *FEN1* is a member of the Flap endonucleases family that encodes a protein characterized as a 5′-specific endonuclease, but its function is also associated with exonuclease activity [70]. As an endonuclease, FEN1 specifically recognizes a double-stranded DNA with a 5′-unannealed flap and makes an endonucleolytic cleavage at the base of the flap [70]. As a 5′ exonuclease, the enzyme progressively degrades nucleotides from a nick or a gap. FEN1 still functions as a critical enzyme in the lagging-DNA strand during the long-path BER (base excision repair) [70]. Recent studies demonstrated that FEN1 also plays a role in apoptosis and cell cycle control [70,71]. In the present study, a tendency of transcript repression of the *FEN1* gene was observed in lymphocytes from AD patients, and this observation deserves further study, considering the important role of this gene in the BER pathway, as well as in the maintenance of genome stability.

Interestingly, the transcript expression of *ATM* and *ATR* was not significantly changed in AD patients (Figure 3). These genes are involved in DNA damage responses, encoding proteins that belong to the PI3K (phosphatidylinositol-kinase) family, which act as sensors of induced DNA lesions and can be activated in response to DNA damage [72–74].

**Figure 4:**
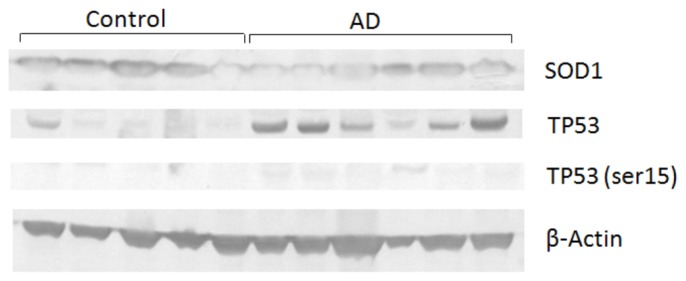
TP53 and SOD1 expression in lymphocytes of AD patients and age-matched control subjects. Protein extraction was performed with the TRIzol reagent, according to the manufacture’s protocol. Western blot was achieved with anti-SOD1, anti-phospho-Ser15-TP53 and anti-TP53. β-Actin was used as an endogenous control.

Moreover, the expression of TP53 and SOD1 proteins was analyzed by Western blot, using β-Actin as an endogenous control (Figure 4). A slight decrease of SOD1 levels in lymphocytes from AD patients was detected. Interestingly, we found increased expression of TP53 in AD patients, in contrast with low levels of TP53-phospho-Ser15 (Figure 4). In spite of the limitation imposed by the low number of patients, TP53 expression profiles observed in this work are in agreement with the fact that *ATM* and *ATR* (responsible for the phosphorylation of TP53 at serine 15) were not significantly upregulated in the present study. The TP53 protein responds to a variety of cellular stresses, regulating target genes that induce cell cycle arrest and apoptosis, senescence and DNA repair, among other responses [75,76]. There are reports showing that TP53 can directly regulate more than 125 genes [77,78], and recent genome-wide chromatin immunoprecipitation studies have revealed that TP53 binds to thousands of targets [75]. This protein triggers apoptosis via intrinsic or extrinsic signaling pathways, which converge to caspase activation, but differ in terms of upstream stimuli [75] and can be related with the increased apoptosis process in neurons (and even in lymphocytes) of AD patients [79]. According to Culmsee *et al.* [80], *TP53* is a candidate gene as a biomarker of AD, being associated with neurodegenerative processes. Uberti *et al.* [81] demonstrated a specific alteration of an intracellular pathway involved in sensing and repairing DNA damage in peripheral cells from AD patients, underscoring the importance of studying this pathway. Furthermore, *CDKN1A* gene (which can be regulated by TP53) was found upregulated in these patients. These results show an important pathway altered in peripheral lymphocytes of AD patients, which deserves further studies.

Western blot analysis even showed a slight decrease in SOD1 levels in lymphocytes from AD patients (Figure 4); this is consistent with the fact that this protein seems to be the main target of oxidative stress in the brain of AD patients [43]. SOD1 is an antioxidant enzyme that plays an important role in the catalysis of superoxide radicals [82], and its deregulation may affect the prevention of oxidative damage.

Cuevas *et al.* [83] showed that AD can be a systemic disease, despite the fact that it has more complex critical neurological alterations. Among the non-neuronal cell function alterations in AD patients, the abnormalities in the immune system were found to be more prominent, and alterations reported in lymphocytes of AD patients could highlight an immunological component characteristic in the pathogenesis of the disease [83]. Therefore, apart from neurological alterations, DNA repair pathways seem to be affected in lymphocytes of AD patients, underscoring its importance in the context of the pathophysiology of AD as a potential biomarker assessment.

## 3. Experimental Section

### 3.1. Subjects

For the comet assay and expression analysis by qPCR, blood samples were collected from 13 patients (AD group), aged between 65 and 90 years old (mean 79.9; standard deviation: ±5.1) (Table 2). Patients were selected according to the DSM-IV (Diagnostic and Statistical Manual of Mental Disorders 4th edition) [84] and the NICDS-ADRDA (National Institute of Neurological and Communicative Disorders and Stroke and The Alzheimer’s disease Related disorders Association) criteria [85]. All AD patients of the present study are sporadic, since they are late onset cases diagnosed over the age of 65. For controls, we selected 14 elderly age-matched healthy controls (EC group), with normal memory and cognitive capacities, with ages between 65 and 90 years (mean ± 73.9; standard deviation: ±5.3) (Table 2). AD and EC underwent a series of tests to exclude dysfunction of kidney, liver, hematologic or thyroid and also syphilis or HIV. Patients with inflammation, neoplasias or smokers were also excluded. Furthermore, on the basis of the routine clinical evaluation, we excluded another kind of dementia, and only patients with a Hachinski isquemic score less than 4 were included in the study.

For Western blot analysis, protein samples were taken from another group of patients (different from the group studied for comet assay and qPCR); this group comprised 6 AD patients (all females, mean age = 78 years) and 5 EC women (mean age = 76 years) selected according to the same aforementioned criteria.

The local ethical committee had approved the research project, and informed consent was obtained for all individuals who participated in this study.

### 3.2. Blood Collection

Peripheral blood mononuclear cells (PBMCs) were isolated from the whole blood by gradient density using Ficoll (Biochrom KG, Berlin, Germany) and immediately submitted to RNA and protein extraction with the TRIzol reagent (Invitrogen, Carlsbad, CA, USA) for further analysis by Western blot and qRT-PCR. For the evaluation of DNA damage by the comet assay, lymphocytes were cultured for 48 h in RPMI 1640 medium supplemented with 20% fetal bovine serum plus Phytohemagglutinin (Invitrogen Corporation, Grand Island, NY, USA).

### 3.3. Cell Culture and Comet Assay

The comet assay was performed to analyze DNA damage and repair kinetics in lymphocytes from the AD (*n =* 8: AD1, AD2, AD3 AD4, AD5, AD9, AD10, AD13; mean age = 81 ± 5.5 ) and the EC group (*n* = 8: EC1, EC2, EC3, EC4, EC5, EC6, EC9, EC14; mean age = 73.7 ± 5.8) cultured for 47 h, followed by treatment with 80 μM hydrogen peroxide (H_2_O_2_) for 1 h; cells were then analyzed at different recovery times: 0, 0.5, 2 and 6 h. Aliquots from each culture were analyzed to check cellular viability (Trypan- Bio-Rad, UK), before performing the alkaline comet assay [86]. This method was carried out with few alterations to detect specific oxidative DNA damage. Since the hOGG1 enzyme, a lesion-specific endonuclease, is capable of identifying the oxidized purine 8OHdG at the DNA molecule, this principle was used in the comet assay to detect oxidized purines [87]. hOGG1 enzyme removes the oxidized purine, causing a DNA strand break, which can be detected by the comet assay. Four replicate slides were prepared for each sample (2 slides were not treated with the enzyme, while another 2 were treated with hOGG1 enzyme). Slides were prepared with 0.5% of normal melting agarose. Then, 40 μL of each sample mixed with 0.5% low-melting point agarose kept at 37 °C was added to pre-coated slides. After gel solidification, slides were immersed, for at least 12 h, in cold lysing solution (2.5 M NaCl, 100 mMEDTA, 10 mMTris Base, pH 10 adjusted with NaOH, to which 1% TritonX-100 and 10% DMSO were freshly added). After lysis, slides were washed and equilibrated in enzyme buffer (40 mMHEPES, 100 mMKCl, 0.5 mM EDTA, 0.2mg/ml BSA; pH 8, adjusted with NaOH). Each slide was then treated with 50 μL of enzyme buffer hOGG1 (1:105 dilution in enzyme buffer, *i.e.*, 0.12 units per gel—New England Bio Labs Inc., Ipswich, MA, EUA) and incubated in a moist box at 37 °C for 20 min. Then, slides were randomly placed in an electrophoresis tank previously filled with fresh electrophoresis solution (1 mM EDTA and 30 0 mM NaOH, pH > 13) and left in the solution for 20 min to allow DNA unwinding and expression of single-strand breaks and alkali-labile sites. Next, electrophoresis was conducted at 4 °C for 20 min, with an electric current of 0.7 V/cm (25 V/300 mA). After electrophoresis, slides were neutralized (0.4 M Tris, pH 7.5 solution) and gels were dehydrated by immersing in absolute ethanol.

The slides were stained with 50 microliters of SYBR Green (2X) and covered with coverslips. The analysis was carried out in a fluorescence microscope (HBO50—Axiolab, Carl Zeiss) equipped with (515–560 nm and 590 nm) barrier filters, connected through a computer. DNA damage was analyzed by the Comet Assay IV software (Perceptive Inst., Bury St Edmunds, Suffolk, UK) which provides the values of tail intensities. Fifty cells per slide were analyzed.

### 3.4. Total RNA and Protein Extraction

RNA extraction from PBMCs was performed with the TRIzol reagent (Invitrogen, Carlsbad, CA, USA), according to the manufacturer’s instructions, immediately after their separation from whole blood samples. RNA integrity was evaluated by denaturing agarose gel electrophoresis under standard conditions.

### 3.5. Quantitative Real-Time PCR (qPCR)

Quantitative real-time PCR was performed in PBMCs from AD patients (*n =* 7: AD2, AD4, AD6 AD7, AD8, AD11, AD12, mean age = 80.6 ± 5.8) and EC individuals (*n =* 7: EC4, EC7, EC8, EC10, EC11, EC12, EC13, mean age = 73.6 ± 5) to evaluate the expression profiles of the following genes: *ATM*, *ATR*, *FANCG*, *FEN1*, *MTH1* and *CDKN1A*. The RNA samples (1 μg) were submitted to reverse transcription reaction using a SuperScript^®^ III Reverse Transcriptase kit (Invitrogen, Carlsbad, CA, USA), according to manufacturer’s instructions. The integrity of cDNA samples was assessed by amplification of the reference *B2M* gene and its visualization in agarose gel electrophoresis. The stability of a panel of four potential reference genes was tested (*TBP*, *HPRT1*, *GUSB*, *B2M*) in control and AD samples using the geNorm software [88]. Based on this analysis, the expression levels of all target genes were normalized by the geometric mean of *HPRT1*, *GUSB*, *B2M*. Primer sequences were designed in exon-exon junction regions to prevent genomic amplification, using the Artemis software v11.4.1 [89] and Primer3 tool v2.2.3 [90]. Amplification efficiencies that were used to calculate the fold-change values were confirmed through standard curves (data not shown). Primer sequences are available in Table 3. Quantitative real-time PCR reactions were performed using a SYBR^®^ Green PCR Master Mix kit (Applied Biosystems, Foster City, CA, USA) in 96-well plates (MicroAmp^®^ Optical 96 Well Reaction Plate-Applied Biosystems, Foster City, CA, USA), according to the manufacturer’s instructions. Standardization of primer and cDNA concentrations were achieved by following the manufacturer’s recommendation. The reactions were carried out with 80 ng of cDNA. Amplification and detection were performed in the ABI PRISM^®^ 7000 Sequence Detection System (Applied Biosystems, Foster City, CA, USA). The expression values were normalized by geometric means obtained for the three reference genes, and expression levels for the groups of individuals were analyzed by the REST 2009 Software-Qiagen [91], using respective reaction efficiencies and 2000 interactions. Significant difference was considered for *p <* 0.05, as estimated by the REST2009 software.

### 3.6. Western Blot

SOD1, TP53 and phospho-Ser15-TP53 protein expression was analyzed by Western blot. All materials for Western blot were purchased from Invitrogen (Carlsbad, CA, USA). Samples (*n =* 6 AD; 5 EC) were prepared from 40 μg of total protein. Protein concentrations were determined using BCA Protein Assay Reagents (Thermo Fisher Scientific Inc., Rockford, IL, USA), according to the manufacturer’s instructions. Proteins were separated by electrophoresis in NuPAGE 4%–12% Bis-Tris gel (Invitrogen, Carlsbad, CA, USA) and blotted onto a PVDF membrane (Invitrogen, Carlsbad, CA, USA). Samples were incubated in blocking buffer for 1 hour before the addition of the primary antibody. The membrane was probed firstly with the primary antibody for SOD1 (1:1,000; Cell Signaling Technology, Beverly, MA, USA) or TP53 (1:1,000; Cell Signaling Technology, USA) or Phospho-TP53(Ser15) (1:1,000; Cell Signaling Technology, Beverly, MA, USA), all of them incubated overnight and then incubated with the secondary antibody for 30 min (Western Breeze Chromogenic Kit, Invitrogen, Carlsbad, CA, USA). Anti-β-actin antibody (1:1,000; Cell Signaling Technology, Beverly, MA, USA) was used as an endogenous control. The immuno-detection was accomplished using a Western Breeze Chromogenic Kit (Invitrogen, Carlsbad, CA, USA). The band intensity was determined using the Scion Image software.

### 3.7. Statistical Analysis

DNA damage evaluated by the comet assay was measured as tail intensities (% of DNA in the tail). The results were analyzed by the SigmaStat software (Jandel Scientific Software, San Jose, CA, USA). The DNA damage was expressed as the mean ± SD (standard derivation). To verify significant differences between groups, analysis of variance (ANOVA) and the *t*-test were applied, followed by a complementary test (pairwise multiple comparisons). Linear regression analysis was applied using GraphPad Prism 4 to examine whether a linear time–response curve could be observed. Significant differences were considered for *p <* 0.05. For the expression data obtained by RT-qPCR, the statistical analysis was carried out by the REST 2009 software using a randomization analysis.

## 4. Conclusions

In the present study, in spite of the low number of individuals analyzed mainly due to the rigorous exclusion criteria, the results support the hypothesis that repair pathways might be compromised in AD. Furthermore, the alterations found in peripheral lymphocytes of AD patients showed that these cells may somewhat reveal some peculiar systemic characteristics of the disease, encouraging further investigation to search for biomarkers present in lymphocytes that might characterize the disease and may provide useful future clinical applications.

## Figures and Tables

**Table 1:** Mean values (±standard deviation) of tail intensities obtained by the comet assay in lymphocytes of patients with Alzheimer’s disease (AD) and elderly healthy controls (EC); cells were cultured for 48 h, treated with H_2_O_2_ during the last 60 min and harvested at different recovery times (0, 0.5, 2 and 6 h).

Collection time (h)	Tail intensity (Alkaline comet assay)			

	AD		EC	
	
	Control	H_2_O_2_	Control	H_2_O_2_
0	8.96 (±4.57)	38.40 (±12.35)	22.73 (±11.73)	41.99 (±7.37)

0.5	9.14 (±4.13)	39.99 (±18.04)	28.97 (±9.60)	37.04 (±17.26)

2	9.08 (±4.79)	31.08 (±15.07)	21.30 (±7.88)	32.30 (±10.44)

6	7.74 (±4.54)	20.93 (±13.16)	21.70 (±9.09)	25.08 (±14.32)

**Collection time (h)**	**Tail intensity (Alkaline comet assay-hOGG1)**			

	**AD**		**EC**	
	
	**Control**	**H_2_O_2_**	**Control**	**H_2_O_2_**

0	17.32 (±10.50)	44.47 (±10.35)	24.10 (±11.12)	46.72 (±18.98)

0.5	13.47 (±5.94)	44.31 (±11.81)	28.64 (±7.88)	51.65 (± 20.33)

2	12.98 (±7.08)	43.90 (±11.89)	34.65 (±21.20)	53.26 (±21.99)

6	15.70 (±8.19)	31.59 (±11.57)	27.85 (±17.29)	46.84 (±20.66)

**Table 2:** Characterization of AD and EC patients, including age, clinical dementia rate (CDR) that identifies the stage of the disease: mild (1), moderate (2) or severe (3); mini-mental state examination score (MMSE): a method for grading the cognitive state of patients (0–30) and gender (M; male; F: female).

AD				
Sample	Age	MMSE	CDR	GENDER
AD 01	79	3	2	M
AD 02	83	3	3	M
AD 03	77	18	1	F
AD 04	90	14	1	F
AD 05	81	2	3	F
AD 06	79	23	–	F
AD 07	72	17	1	M
AD 08	76	9	3	F
AD 09	86	20	2	F
AD 10	72	14	2	F
AD 11	80	15	2	F
AD 12	84	–	1	F
AD 13	80	14	1	F
**EC**				
**Sample**	**Age**	**MMSE**	**CDR**	**GENDER**
EC 01	86	–	–	F
EC 02	69	–	–	F
EC 03	72	–	–	M
EC 04	70	–	–	F
EC 05	72	–	–	F
EC 06	69	–	–	F
EC 07	76	–	–	F
EC 08	74	–	–	F
EC 09	78	–	–	F
EC 10	70	–	–	F
EC 11	74	–	–	F
EC 12	68	–	–	F
EC 13	83	–	–	F
EC 14	74	–	–	M

**Table 3:** Forward and reverse primer sets designed for genes analyzed by RT-qPCR.

Primers	Sequence	Product size (pb)
*ATM–forward*	5′–GACGTTACATGAGCCAGCAA–3′	100
*ATM–reverse*	5′–CACATGCGATGGAAAATGAG–3′	
*ATR–forward*	5′–GTGAGTGGAAGCCATGAGG–3′	109
*ATR–reverse*	5′–ACAAATGACAGGAGGGAGTTG–3′	
*FEN1–forward*	5′–ATTCCCATGGCAACACAGAG–3′	112
*FEN1–reverse*	5′–AGGGAGAGCGAGCTTAGGAC–3′	
*MTH1–forward*	5′–CGTGGAGAGCGACGAAAT–3′	103
*MTH1–reverse*	5′–CTGAAGCAGGAGTGGAAACC–3′	
*P21–forward*	5′–CTTCCTGTGGGCGGATTAG–3′	105
*P21–reverse*	5′–GACTCTCAGGGTCGAAAACG–3′	
*FANCG–forward*	5′–GACAGCAGTTGGCTCAGGAT–3′	102
*FANCG–reverse*	5′–CAGTCAGCTCCAAGGGAAGA–3′	
*B2M–forward*	5′–AGGCTATCCAGCGTACTCCA–3′	112
*B2M–reverse*	5′–TCAATGTCGGATGGATGAAA–3′	
*GUSB–forward*	5′–CACCAGGATCCACCTCTGAT–3′	115
*GUSB–reverse*	5′–TCCAAATGAGCTCTCCAACC–3′	
*HPRT1–forward*	5′–TCATTATGCTGAGGATTTGGA–3′	104
*HPRT1–reverse*	5′–GATGGCCTCCCATCTCCTT–3′	
*TBP–forward*	5′–AGGAGCCAAGAGTGAAGAACAG–3′	117
*TBP–reverse*	5′–CTCCCCACCATGTTCTGAAT–3′	
